# A bibliometric analysis of the literature on *Mycoplasma* and *Ureaplasma* infections

**DOI:** 10.4102/sajid.v41i1.788

**Published:** 2026-02-28

**Authors:** Winnie T. Ramaloko, Samson A. Malgwi, Sinethemba H. Yakobi, Matthew A. Adeleke, Nathlee S. Abbai, John Osei Sekyere, Nontuthuko E. Maningi

**Affiliations:** 1 Department of Microbiology, Faculty of Agriculture, Engineering and Science, University of KwaZulu-Natal, Durban, South Africa; 2 Department of Genetics, Faculty of Agriculture, Engineering and Science, University of KwaZulu-Natal, Durban, South Africa; 3 Department of Clinical Medicine, Faculty of Health Sciences, University of KwaZulu-Natal, Durban, South Africa; 4 Department of Clinical Development, Medical diagnostic Laboratories, Genesis Biotechnology Group, Trenton, New Jersey, United States of America; 5 Department of Medical Microbiology, Faculty of Health Sciences, University of Pretoria, Pretoria, South Africa

**Keywords:** bibliometric analysis, *Mycoplasma*, *Ureaplasma*, infections, asymptomatic

## Abstract

**Background:**

*Mycoplasma* and *Ureaplasma* species are understudied opportunistic pathogens that infect humans, animals and plants. These infections are often asymptomatic, which, together with fastidious growth requirements, makes them challenging to detect.

**Aim:**

The review aimed to provide a bibliometric analysis of available literature to reflect and assess trends, progress and knowledge gaps in this field.

**Setting:**

This article is a literature review.

**Method:**

A bibliometric analysis of 19 486 documents from 1992 to 2022 was conducted using the Web of Science database and in-depth analyses on RStudio.

**Results:**

China and the United States produced a high number of publications and citations. South Africa, the first most-cited African country, contributed 685 publications, ranking 24th globally with 2031 citations. *Veterinary Microbiology* was the highest performing journal with 448 papers and 10 036 citations. The most frequent keywords were ‘infection’ and ‘*Ureaplasma urealyticum*’.

**Conclusion:**

Research on *Mycoplasma and Ureaplasma* infections has progressed over time, but mainly in developed countries. The restricted publications and moderate citations in South Africa suggest research gaps in understanding the true burden and impacts of these infections.

**Contribution:**

This study provides a comprehensive bibliometric overview of *Mycoplasma and Ureaplasma* infections and reports the global and local progress in this field. The overall moderate, steady growth highlights the need for broader international collaboration and expanded research efforts in low-resource countries to address existing research gaps. This expansion is essential, particularly in clinical research, for strengthening both surveillance and treatment guidelines.

## Introduction

Mycoplasmas, including *Mycoplasma* and *Ureaplasma* species, are free-living, self-replicating, small microorganisms classified as Mollicutes.^1^ Mollicutes have eight genera and over 200 known species identified from humans, plants and animals.^2^ In humans, mycoplasmas occur on the epithelial surfaces of respiratory and urogenital tissues.^3^ They infect the phloem sieve in plants^4^ and are found on mammary glands and serous membranes in animals.^3,5^ *Mycoplasma* and *Ureaplasma* spp. cause symptomatic and asymptomatic infections. In humans, urogenital *Mycoplasma* and *Ureaplasma* usually cause asymptomatic infections and coexist with other sexually transmitted infections (STIs).^6,7^ They are transmitted through direct genital contact, and if untreated, may result in chronic and acute non-gonococcal urethritis (NGU) in males^8^ and ectopic pregnancy, cervicitis, infertility and pelvic inflammatory diseases (PIDs) in women.^8,9,10^ *Mycoplasma pneumoniae* is the most common pathogen among mycoplasmas and the only respiratory mycoplasma thus far. It causes community-acquired pneumonia (CAP), mostly in children, accounting for 41.1% of paediatric CAP cases.^11,12,13^ *Mycoplasma* and *Ureaplasma* infections are not fully explored. Therefore, a bibliometric study will help unpack the patterns and gaps in this topic and possibly predict prospects for future research.

Bibliometric analysis uses bibliographic data and statistically assesses published papers to detect and understand their connections.^14^ Furthermore, this analysis can examine patterns and developments in the field of research. This method traces the conceptual history of a particular area of study and predicts upcoming trends. In addition to making huge dataset analysis easier, it enables carrying out a more systematic literature study, gathering data and spotting trends. By considering what has been effective in previous papers, these data can be utilised to enhance the quality of future documents. Ultimately, it can potentially enhance the discoverability of research papers.

Bibliometric analysis on various infectious diseases has been done to predict the patterns and burden of infections globally. Although *Mycoplasma* and *Ureaplasma* spp. have been reported for years, to our knowledge, no bibliometric analyses have been undertaken. Therefore, this study presents the first bibliometric analysis on *Mycoplasma* and *Ureaplasma* infections. The study’s main objectives were to evaluate the global research trends of available literature on *Mycoplasma* and *Ureaplasma* infections over three decades. To achieve this, we analysed (1) the annual number of publications, (2) the total and mean number of citations, (3) the most influential sources, and (4) the most frequently used keywords using conceptual mapping.

## Methods

### Literature search strategy and data collection

**FIGURE 1:**
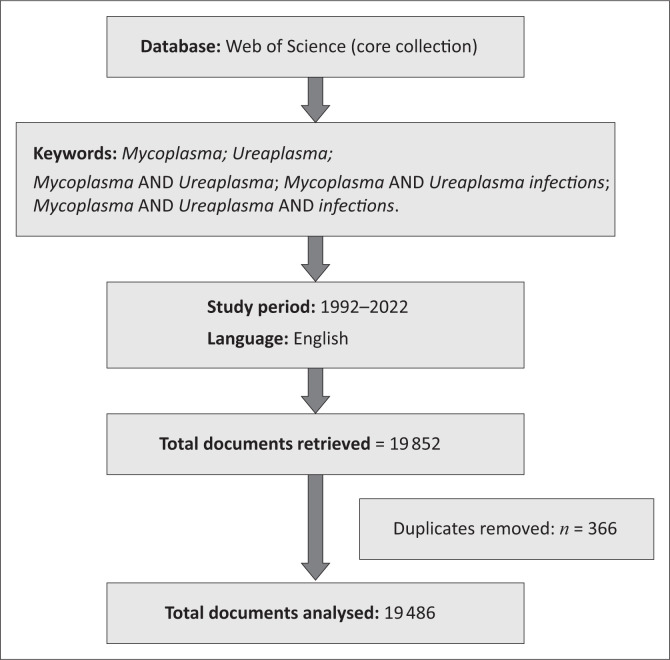
A flow diagram outlining the literature search strategy.

We searched the Web of Science (Core Collection) database from the start of 1992 until the end of 2022. This database has numerous advantages, including a robust citation index and a comprehensive history that allows researchers to explore research trends easily. We focused on peer-reviewed documents only. The search was performed using the keywords ‘*Mycoplasma, Ureaplasma, Mycoplasma* AND *Ureaplasma, Mycoplasma* AND *Ureaplasma* infections, *Mycoplasma* AND *Ureaplasma* AND infection’ in November 2023. The initial number of documents was refined by applying the following exclusion criteria: removing duplicates, removing documents published before 1992 and documents not published in English. The initial search resulted in 19 852 documents. For the past three decades (1992–2022), after 366 duplicates were removed, 19 486 documents were available for bibliometric analysis (Figure 1). BibTeX files of included documents were extracted from the Web of Science for further analyses using the R software.

### Data analysis

Data analysis, including information extraction and data visualisation, was done using Bibliometrix analysis^15^ and Biblioshiny software in R package (RStudio Posit Software, PBC [formerly RStudio]; Boston; Massachusetts, United States [US]).^16^ Countries’ performance, citation performance, collaborations, relevant journals and frequent keywords were analysed. This analysis presents the scientific production, citations, citable years (number of years a publication is publicly available and eligible for citation), ranking of countries by the number of publications, as well as the most cited journal, from 1992 to 2022. Journal g- and Hirsch index (h-indexes) were analysed and assessed. The g- and h-index look at the impact of research through productivity and citations. The generalised h-index (g-index) looks at the number of published documents and their citations, while the h-index represents the distribution of the most highly cited documents.^17^ Finally, the most frequently used keywords (such as KeyWords Plus and Author keywords) in this research topic, along with their co-occurrence, were assessed using conceptual maps, including Co-Word Network and thematic maps. KeyWords Plus (ID) are fixed words (not selected by the author) generated from cited titles that frequently appear in the titles of an article’s references, but do not appear in the title of the article itself. Also, this is a word and/or phrase and/or terms dictionary ranked based on the Web of Science databases’ algorithm. The ID could improve the efficiency of cited-reference searching, whereas Author’s Keywords (DE) are specific descriptors selected by the authors in published articles. This assists readers in indexing and finding research towards discovering fundamental concepts beyond the article’s title or abstract. The total number of documents analysed served as the denominator for each calculation: multi-authored documents, national co-authorship and international co-authorship. Data tables and visuals for the retrieved documents were also performed on Microsoft Excel (https://office.microsoft.com/excel), and the final image processing was done on Paint.NET (https://www.getpaint.net/).

### Ethical considerations

This article followed all ethical standards for research without direct contact with human or animal subjects.

## Review findings

### Literature search, data collection and analysis

Relevant literature was searched and extracted from the Web of Science (Core Collection) database in November 2023. A total of 19 486 documents, published between 1992 and 2022 in the English language, were extracted, and after 366 duplicates were removed, our study sample comprised 19 486 publications (Figure 1).

Key findings, shown in Table 1, included the general growth rate of publications, an overview of scientific outputs, sources, citation performance, authors and collaborations.

**TABLE 1:** Summary of key information of the scientific documents on *Mycoplasma* and *Ureaplasma* infections (*N* = 19 486).

Description	Results
Documents	19 486
Timespan	1992–2022
Sources (journals, books, etc.)	2455
Annual growth rate (%)	4.2
Average citations per document	26.6
**Document content**	
Keywords plus (ID)	17 505
Author’s keywords (DE)	19 563
**Authors**	
Authors	49 776
Authors of single-authored documents	437
**Authors’ collaborations**	
Single-authored documents	596
Co-authors per document	5.7
International co-authorship %	19.8

ID, system-generated keywords; DE, author’s keywords.

A total of 2455 sources contributed to the document publication, with an average citation of 26.6 per document.

An impressive number of 49 776 authors contributed to these publications, 437 of whom were single authors across 596 documents. For documents with several authors, the percentage of international co-authorship was 19.8%, which represents collaboration between authors from different countries. The overall annual growth rate for articles about *Mycoplasma* and *Ureaplasma* infections was 4.2%, indicating a moderate growth rate.

This was determined from the initial number of documents (in 1992) and the final number of documents (in 2022).

### Global scientific documents production

**FIGURE 2:**
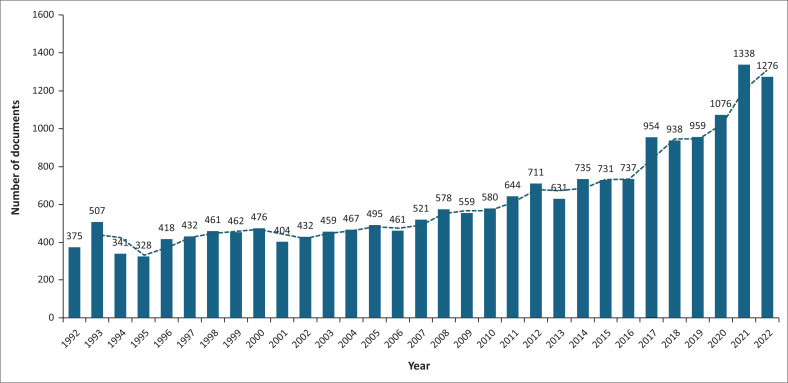
The annual publication growth on *Mycoplasma* and *Ureaplasma* infections from 1992 to 2022.

The annual production of scientific documents is shown in Figure 2.

Overall, there was an increase in the production of scientific documents on *Mycoplasma* and *Ureaplasma* infections. The publication output increased steadily, with more than 300 documents published annually between 1992 and 2010, followed by a sustained increase to over 600 publications per year in subsequent years. Notably, the last 2 years of the study period (2021–2022) showed a pronounced surge in research output, with annual publications exceeding 1200 documents. This sharp increase indicates the highest level of scientific productivity observed across the entire timespan and shows an accelerated growth.

### Most productive countries and their citation trends

To evaluate each country’s contributions, publications were analysed based on document production growth rate and their total citations (TC). The list of all contributing countries and their citation profiles is summarised in Table 1-A1 Online Appendix 1. Of the 141 contributing countries, 91 countries (64.5%) produced fewer than 100 documents since 1992. Furthermore, this category is comprised mostly of developing countries, particularly from Africa. South Africa was ranked 24th with 685 documents published over 30 years (Table 1-A1 Online Appendix 1).

**FIGURE 3:**
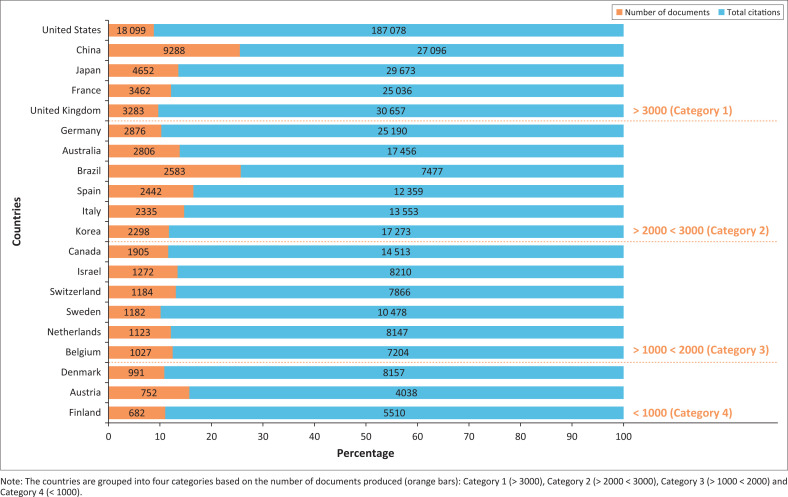
Document production and citations of the top 20 countries between 1992 and 2022.

The overall document production and TC for the top 20 countries are shown in Figure 3. Contributing countries were divided into four categories based on publication numbers from 1992 (Figure 3).

The United States led global research output, followed by China and Japan, reflecting long-term investment in infectious disease research, established scientific infrastructure and funding. Germany and Australia were prominent in Category 2, while Canada led Category 3. Countries in the second and third categories demonstrate notable research capacity. Although lower in volumes than the leading countries, they still make substantial contributions to the field. Collectively, this categorisation shows global disparities in research productivity while also identifying countries with emerging or moderate research capacity that contribute significantly to the advancement of knowledge on *Mycoplasma* and *Ureaplasma* infections.

Specifically, global production was led by the United States, with 18 099 documents produced since 1992, followed by China (*n* = 9288) and Japan (*n* = 4652) documents published. The United Kingdom (UK) showed the least document production in this top category (*n* = 3283).

### Global citation trends based on research on *Mycoplasma* and *Ureaplasma* infections

Citations in research are intended to establish the ancient knowledge of a particular research topic. They make substantial contributions to pushing forward the boundaries of knowledge.^18^ The TC and the average document citations of various countries are shown in Table 2-A1 Online Appendix 1. The United States presented the highest TC (*n* = 187 078) for the 18 099 produced documents, with an average document citation of 41.4. The UK and Japan were ranked second and third in TC, presenting 30 657 and 29 673 TC, respectively. These countries presented the average document citations of 34.3 (UK) and 25.6 (Japan). Although Sweden produced fewer documents (*n* = 1182) with a TC of 10 478, it obtained an impressive average document citation of 37.4. The same trend was observed in Finland, with an average document citation of 37.5 from 682 publications and 5510 TC. Nonetheless, Finland was part of the top 20 performing countries. Moreover, China, the second most performing country in document publications (*n* = 9288), obtained the lowest average document citation of 12.4. However, this country was ranked number four based on the total document citations from 1992 to 2022 (Table 2-A1 Online Appendix 1). In Africa, South Africa (SA) received the most citations (*n* = 2031), followed by Tunisia with 1581 TC; however, this regional lead is still lower than that of the United States, which produced the highest number of citations globally (*n* = 187 078).

The annual mean citation, mean citations per document and the citation years are shown in Table 3-A1 Online Appendix 1. The mean citations per document represent the average number of times a document has been cited, thus measuring the long-term cumulative impact. The annual mean citations represent the total cumulative mean citations for each year. The highest mean citation was detected in 2006 (3.0), with a mean document citation of 39.9 in 19 years, followed by 2015 (2.7), 30.0 in 10 years. The lowest mean citation was observed in 2022 (0.6), with a mean document citation of 2.4 across three citable years. These citation matrices show that new publications have a smaller citation window compared to older publications, which have longer exposure. Even so, the year 1992, with the highest number of citable years (*n* = 33), had a lower annual mean citation (1.1) and a mean citation per document (37.1), as compared to the peak years (i.e. 2006 and 2015) with fewer citable years.

### Top-performing journals

The top 20 list of contributing journals is shown in Table 4-A2 Online Appendix 2. A total of 2455 journals contributed to the research on *Mycoplasma* and *Ureaplasma* infections. Of the 2455 journals, 30 (1.2%) published over 100 documents, and 84.1% (*n* = 2064) of journals published less than 10 documents since 1992. Of the sources with fewer than 10 documents, 43% (*n* = 1056) published only one document from 1992 to 2023 (Table 4-A2 Online Appendix 2).

#### Journal citations and their impact

The top 20 journals that produced documents on *Mycoplasma* and *Ureaplasma* infections are shown in Table 2.

**TABLE 2:** The top 20 most influential journals publishing research on *Mycoplasma* and *Ureaplasma* infections.

Journal	h-index	g-index	TC	P (*n*)	PY_start
*American Journal of Obstetrics and Gynecology*	91	146	24 634	256	1992
*Journal of Clinical Microbiology*	76	106	18 998	410	1992
*Clinical Infectious Diseases*	66	103	14 607	291	1992
*Infection and Immunity*	56	81	11 179	292	1992
*Antimicrobial Agents and Chemotherapy*	53	76	8960	230	1992
*Journal of Bacteriology*	49	79	8368	199	1992
*PLOS One*	49	78	10 978	391	2007
*Veterinary Microbiology*	49	70	11 458	450	1992
*Proceedings of the National Academy of Sciences of the United States of America*	43	56	6474	56	1992
*Journal of Infectious Diseases*	42	73	5587	90	1992
*Pediatric Infectious Disease Journal*	41	68	6179	176	1992
*Sexually Transmitted Diseases*	40	55	3889	138	1993
*Sexually Transmitted Infections*	40	59	4317	134	1999
*Journal of Maternal-Fetal & Neonatal Medicine*	39	70	5438	134	2005
*Applied and Environmental Microbiology*	38	67	4578	78	1992
*Nucleic Acids Research*	38	78	6238	79	1992
*Obstetrics and Gynecology*	37	59	3804	59	1992
*International Journal of STD & AIDS*	36	50	3824	188	1992
*Journal of Antimicrobial Chemotherapy*	36	49	2802	88	1992
*Acta Obstetricia et Gynecologica Scandinavica*	35	54	3013	59	1993

h-index, Hirsch index; g-index, generalised h-index; TC, total citations; P, publications; PY_start, start of publication year; PLOS, Public Library of Science.

The table displays the h-index, g-index, TC, number of publications and the year of first publication.

The most cited journal was the *American Journal of Obstetrics and Gynecology* with 24 634 citations. This journal also presented a high g-index (146) and h-index (91). This was followed by the *Journal of Clinical Microbiology* and the *Journal of Infection and Immunity* with 18 998 and 11 179 TC, respectively.

The *American Journal of Obstetrics and Gynecology* was the leading channel with 256 published documents in 30 years. These documents were cited 24 634 times with a g-index and h-index of 146 and 91, respectively.

Other top-performing journals with over 10 000 TC included the *Journal of Clinical Microbiology* (*n* = 18 998), *Journal of Clinical Infectious Diseases* (*n* = 14 599), *Journal of Veterinary Microbiology* (*n* = 11 458), *Journal of Infection and Immunity* (*n* = 11 179) and *PLOS One* (*n* = 10 978). All these sources started publishing in the year 1992, except for *PLOS One*, which only started publishing in 2007. *PLOS One* published 391 documents with a g- and h-index of 78 and 49, respectively. The journal with the lowest TC in the top 20 sources was the *Journal Acta Obstetricia et Gynecological Scandinavica*, with 59 published documents and 3804 TC since 1992. This journal presented a low g-index (59) and h-index (37).

### Author collaboration and impact

**FIGURE 4:**
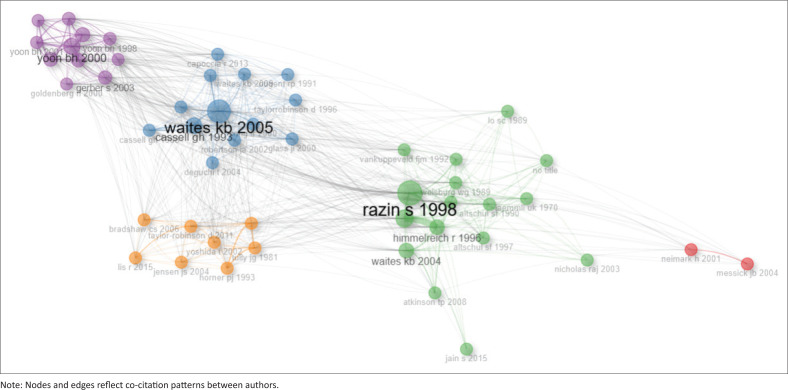
Author co-citation network on publications that focus on *Mycoplasma* and *Ureaplasma* infections.

**FIGURE 5:**
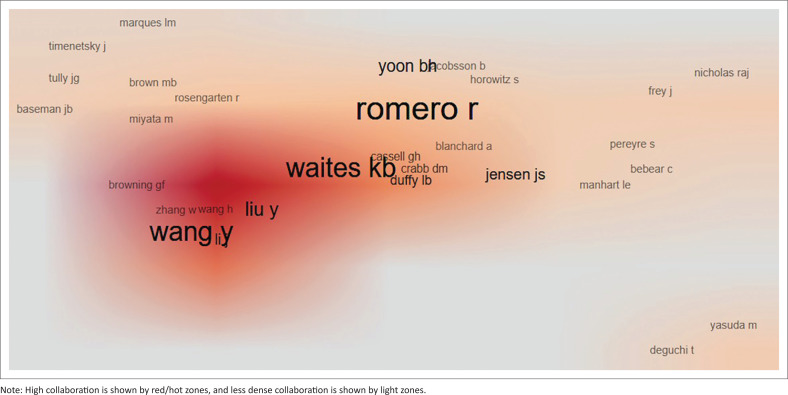
Author collaborative overlay, where heatmap intensity and node size represent collaborative frequency.

The bibliometric analysis of author collaboration and paper impact revealed the most influential authors on *Mycoplasma* and *Ureaplasma* research. The co-citation network mapping shows distinct colour clusters that suggest thematic groupings with strong connections and cross-citations between clusters. Papers such as ‘Waites KB. 2005’^1^ and ‘Razin S. 1998’,^19^ with large nodes, imply their impact and frequent cross-citation within the research field (Figure 4). The author collaboration overlay in Figure 5 shows collaboration density using a heatmap gradient. Authors with a larger and bolder font size indicate high activity and influence. The dark red, orange and whitish regions represent areas of frequent, moderate and low collaborations, respectively. Authors such as Waites KB, Wang Y and Romero R appear to be highly collaborative and influential, with high collaboration observed among Liu Y, Zhang G, Browning GF and Wang H.

### Most frequent keywords

**FIGURE 6:**
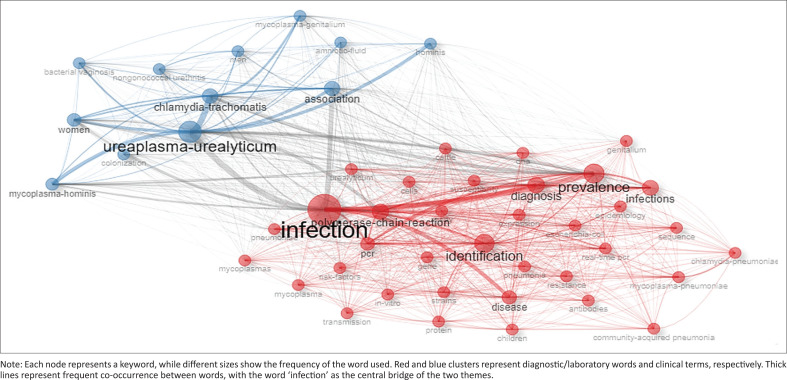
Network visualisation of the co-occurrence of frequently used keywords identified through literature co-occurrence analysis.

Keyword co-occurrence is the phenomenon of multiple keywords in a single article. Such keywords can help identify current trends on different research topics and may also help track changes in those topics.^20^ We found 17 550 frequently used keywords among the 19 486 identified documents. The word ‘infection’ was the most used, appearing 3020 (17.2%) times. The second and third most frequent keywords were ‘*Ureaplasma urealyticum*’, appearing 1691 (9.5%) times, followed by ‘identification’ at 1667 (9.5%). The words ‘prevalence’ (*n* = 1460; 8.31%), ‘diagnosis’ (*n* = 1259, 7.0%) and ‘infections’ (*n* = 1228, 6.9%) also made the top 10 the most common keywords in our research topic. Other common keywords in the top 10 included disease (*n* = 1150), ‘polymerase-chain-reaction’ (*n* = 1137) and ‘association’ (*n* = 1005) (Table 5-A2 Online Appendix 2). Common keywords such as cattle and swine both appeared 349 times, contributing 2%. These keywords represent veterinary studies. Other keywords include *gallisepticum*, pigs, chicken, sheep, *synoviae* and *hyopneumoniae*, which also appeared over 100 times since 1992. A visual representation of the keyword co-occurrence network was produced using Biblioshiny (Figure 6). The most common keywords were categorised into two groups: (1) diagnostic and methodological or laboratory and (2) clinical and epidemiological terms. Theme one encompasses terms related to the detection and technical investigation of pathogens. Terms included identification, diagnosis, diseases, prevalence and polymerase chain reaction. The second cluster focuses on specific organisms, infection-related effects and host populations. Organisms such as *Chlamydia trachomatis* (*n* = 983), *Mycoplasma genitalium* (*n* = 661) and *Ureaplasma urealyticum* (*n* =1691) were the most common terms, along with demographic terms such as women (*n* = 872) and men (*n* = 471).

## Discussion

We analysed bibliometric data on ‘*Mycoplasma* and *Ureaplasma* infections’ from 1992 to 2022. Genital mycoplasmas, particularly *M. genitalium*, are considered clinically important as they cause various STIs and, if left untreated, can lead to serious reproductive health complications in both men and women and adverse pregnancy outcomes. Most developing countries have a high burden of human immunodeficiency virus (HIV) infections.

Therefore, if genital *Mycoplasma* and *Ureaplasma* infections are not investigated and treated, this may lead to an increased risk of HIV transmission and acquisition.

Furthermore, we created visual representations of the trends and current insights on the research topic. These findings could be a point of reference and recommendations for future studies related to *Mycoplasma* and *Ureaplasma* infections. We looked at the main information extracted from the 19 486 documents. We documented a continuous increase in publications since 1992, with a notable growth in the early 2000s, likely because of the adoption of molecular diagnosis rather than culture and increased awareness. The increase in publications observed in the last 2 years of the study period (2021–2022), with more than 1200 documents published annually, suggests recent intensification of research efforts in this field, likely driven by improved molecular diagnostics, heightened awareness of atypical and co-infecting pathogens, increasing research funding and broader global interest in infectious disease surveillance.

Citations and other research outputs in this research topic increase by 4.2% annually, indicating moderate and steady growth. The top three contributing countries based on publications were the United States (*n* = 18 099), China (*n* = 9288) and Japan (*n* = 4652) (Figure 3). These countries also received more citations than other countries.

The United States’ emergence as the leading country in *Mycoplasma* and *Ureaplasma* infections likely indicates a significant study focus because it has more resources and research funding. Moreover, the United States has funding that directly supports multicentre research activities on STIs, including genital mycoplasmas.^21^ Such advantages increase literature visibility, which does not necessarily translate to prevalence. The US government invests in STI research,^21^ as opposed to middle-income and especially low-income developing countries (commonly sub-Saharan African countries like South Africa), where funding is limited, and research depends on aid from other countries.^21,22^ Possible reasons for the United States’ predominance in citations include (1) more research outputs and consequently citations, (2) prestigious research institutions, which may influence the number of citations by receiving more attention, most probably because of quality and groundbreaking research outputs, (3) major database indexing that covers mainly journals from high-income countries. Poor scientific production rates in developing countries may be because of the lack of sustainable funding, lack of resources and infrastructure, few collaborations and insufficient government investment in such research initiatives. In South Africa, 685 publications and 2031 citations were reported since 1992. While this reflects a considerable research activity, the relative citation impact suggests low international visibility in comparison to countries with apparent global impact and visibility. Factors such as diagnostic challenges and no routine surveillance may reduce clinical awareness, as well as the journals where research work is published, which might contribute to the low productivity and citations in developing countries.^23^ South African data on *Mycoplasma* and *Ureaplasma* infections are published frequently in the *Southern African Journal of Infectious Diseases, Sexually Transmitted Infections: Journal of Applied Microbiology* and *The Journal of Infection in Developing Countries*. This is likely the barrier to the production and citations observed in South Africa.

*Mycoplasma* and *Ureaplasma* infections can be asymptomatic, which may add to the topic’s unpopularity because of low diagnosis records. Nevertheless, citations in this research field are quite impressive. The most cited documents (*n* = 731) with a mean TC of 30.04 per document were produced in 2015. These citations could have been driven by *Mycoplasma* outbreaks, particularly for *M. pneumoniae*, which occur seasonally every 3–7 years.^24,25^ Publications between 2004 and 2010 were quite low compared with those after 2020. However, citations between 2004 and 2010 were high, while TC post-2020 were low. As expected, given the increasing annual publication count after 2010, the mean citations per year may also increase as more work is done on this topic. China (*n* = 9288) and the United States (*n* = 187 078) represented the highest publications globally. This indicates that these two countries are leading performers, as evidenced by their TC rates and overall scientific outputs. Citations are also influenced by collaborations, particularly international ones, which accounted for 19.8% in our study. This is also influenced by publishing in journals with a strong impact and an established reputation.

The dataset of publications on *Mycoplasma* and *Ureaplasma* infections is spread across several journals focused primarily on veterinary science, infectious diseases and microbiology. Although journals such as *Clinical Microbiology* (410), *PLOS One* (391) and *Veterinary Microbiology* (450) published more documents, the *American Journal of Obstetrics & Gynecology* had the most TC and is the most influential in our research topic. A similar trend was also observed in an American journal, the *Journal of Proceedings of the National Academy of Sciences* of the United States (US), which produced 6474 citations from 56 documents since 1993. The *Journal* of *Acta Obstetricia et Gynecologica Scandinavica* presented the lowest citations (3013) from the lowest number of publications (59) since 1993. This suggests that articles in this journal do not reach a broader audience of researchers and are therefore less cited or used. Given the evidence that this journal was the lowest in the list and had lower TC than documents, we can safely conclude that the journal was less influential and made fewer contributions to this topic.

Our study analysed documents based on *Mycoplasma* and *Ureaplasma* infections as broad terms and did not arrange documents based on anatomical systems or clinical emphasis. This may not give a full representation of non-reproductive infections caused by clinical *Mycoplasma* and *Ureaplasma* species. Future bibliometric studies should classify keywords by clinical domains, such as urogenital, respiratory and systemic, to get a full representation of the literature.

Keywords from both veterinary and clinical settings were reported. Veterinary keywords suggest that studies on *Mycoplasma* and *Ureaplasma* infections in animals also contribute to the overall growth of this research topic. Nevertheless, keywords in clinical research appear more predominant, as shown in Figure 6. Our analysis of common keyword occurrence indicated that the word ‘infection’ was the leading keyword in this research topic from 1992 (Figure 6 and Table 5-A2 Online Appendix 2). The keyword ‘*Ureaplasma urealyticum*’, which is a bacterium that infects the genital tracts in humans, was the second most frequently used keyword. This may suggest that clinical *Mycoplasma* and *Ureaplasma* infections are the most studied infections. Furthermore, it is not surprising that this word tops the list, as it is the most commonly studied urogenital pathogen in the *Mycoplasma* family because of its commensal nature in the lower urogenital tract of human adults, mostly females.^26^ However, these data were from developed countries, particularly countries outside Africa. This is evident because SA was the leading country in Africa with 685 documents between 1992 and 2022 (Table 6-A2 Online Appendix 2).

Moreover, fewer studies on clinical *Mycoplasma* and *Ureaplasma* infections were reported in South Africa.

Analysis of collaborative patterns, as shown by affiliations and study populations (Online Appendix 1), revealed that co-citations and high collaboration occurred between authors from America, Europe and Asia. Importantly, this analysis showed no South African authors or institutions, suggesting the lack of international collaborations and less engagement on this research topic, signalling the need for more research and collaborations.

All the top 10 frequent keywords co-occur with one another. The keywords ‘infection, identification, prevalence’ may indicate that most studies focused on the prevalence of these infections. Several investigations have revealed the prevalence of *Mycoplasma* and *Ureaplasma* infections.^7,27,28,29,30,31^ Furthermore, keywords such as diagnosis and polymerase-chain-reaction (PCR) suggest that PCR is the most commonly used method for diagnosing *Mycoplasma* and *Ureaplasma*.^27,28,32,33,34^ The terms ‘*Ureaplasma urealyticum*’, ‘association’ and ‘*Chlamydia trachomatis*’ all imply the co-existence of these infections. This is not surprising, given that *Mycoplasma* and *Ureaplasma* infections often co-exist with other STIs, including *C. trachomatis*.^7^ This observation also suggests a frequent co-infection or overlapping research covering both organisms. *Mycoplasma* and *Ureaplasma* spp. usually induce asymptomatic infections and are usually discovered in symptomatic patients with other STIs. Therefore, keywords such as ‘*Mycoplasma*’ and ‘*Ureaplasma*’ mostly co-occur with other STIs or STI-causing pathogens. The limitation of the study is that the analysis was solely based on the Web of Science database, which only focuses on peer-reviewed documents, and this may not have included all relevant literature indexed by other databases.

Additionally, the appearance of keywords that focus on antimicrobial resistance, resistance mechanisms and laboratory diagnostics suggests potential issues to be tackled. Therefore, future studies on genital *Mycoplasma* and *Ureaplasma* infections must be conducted, looking into specified populations, the emergence of antimicrobial resistance, the prevalence of these infections and finding diagnostic markers that can be used to develop new, rapid diagnostic assays. Some studies can be conducted to identify virulence markers to understand the pathogenicity of these species. These studies will guide the development of effective public health policies, such as screening programmes for high-risk populations and screening partners to prevent the continuous spread of these infections, thus bridging the local research gaps.

### Recommendations

Despite the increasing interest in *Mycoplasma* and *Ureaplasma* infection research, further research should focus on integrating infection management approaches as well as expanding the use of molecular diagnostic techniques for enhanced diagnosis. This could further accelerate research innovation, granting access to advanced technologies in *Mycoplasma* and *Ureaplasma* infection diagnosis. The bibliometric analysis reveals the critical need for collaborative research efforts to improve diagnostic technologies as well as lower the cost of diagnostic kits, particularly in low- and middle-income countries, such as South Africa. Local research initiatives and expanding international collaboration should be prioritised in such countries.

## Conclusion

According to our bibliometric analysis on *Mycoplasma* and *Ureaplasma* infections between 1992 and 2022, there has been a noticeable increase in the amount of literature generated over the years. The key findings of the bibliometric metrics showed increasing publications and citations. This increase in research activity could be predicated on improvements in technology, research funding and increasing collaborative activities.
